# Supplementary material for: Carafe2 enables high quality in silico spectral library generation for timsTOF data-independent acquisition proteomics
Source: bioRxiv. 2026 Mar 31:2026.03.27.714846. Preprint. [Version 1] doi: 10.64898/2026.03.27.714846 (PMC13060252; doi:10.64898/2026.03.27.714846)
Supplement: Supplement 1 [file media-1.pdf]

Supplement to "Carafe2 enables high quality *in silico* spectral library generation for timsTOF data-independent acquisition proteomics"

Bo Wen[1], J. Sebastian Paez[2], Chris Hsu[1], Daniele Canzani[2], Alexis Chang[1], Nicholas Shulman[1], Brendan X. MacLean[1], Matthew D. Berg[1], Judit Villén[1], William E. Fondrie[2], Lindsay K. Pino[2], Michael J. MacCoss[*1], and William S. Noble[*1,3]

[1]Department of Genome Sciences, University of Washington
[2]Talus Bioscience, Inc. Seattle, WA, USA
[3]Paul G. Allen School of Computer Science and Engineering, University of Washington

[*]Correspondence: maccoss@uw.edu, wnoble@uw.edu

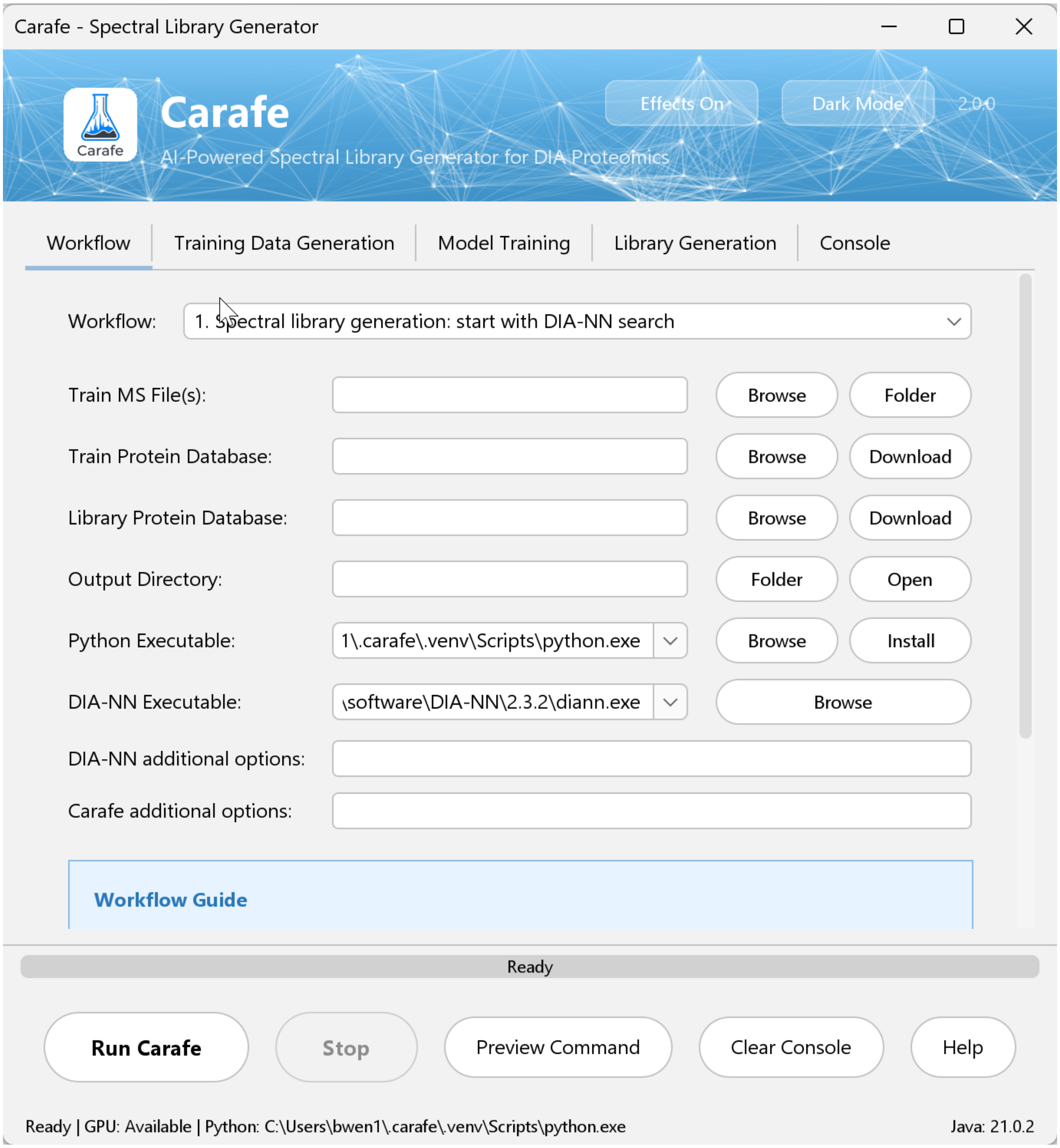

Supplementary Figure 1: **Carafe2 graphical user interface.** The Carafe2 GUI provides an intuitive interface for users to input parameters, select data files, and execute the spectral library generation process.

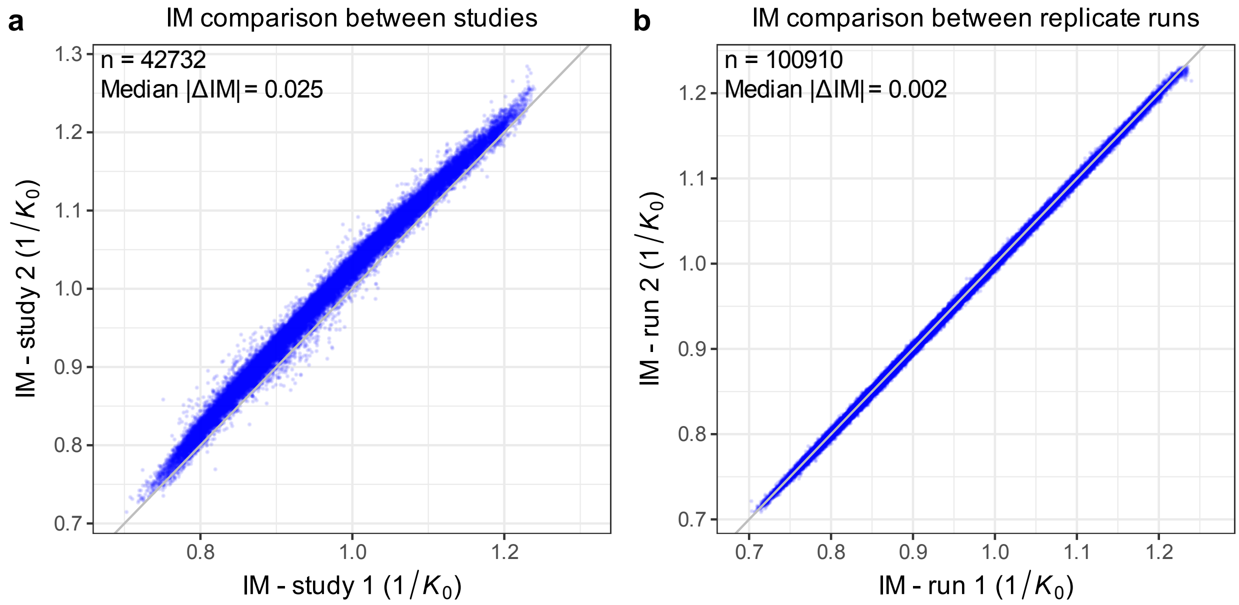

Supplementary Figure 2: **Comparison of ion mobility values between studies and replicate runs.** Scatter plots compare ion mobility values for matched peptide precursors detected in two MS runs. **(a)** Comparison between runs acquired in two independent studies (cross-study). **(b)** Comparison between two replicate runs acquired within the same study (within-study). Each point represents a matched precursor present in both runs; the gray diagonal indicates perfect agreement ($y = x$). Points are colored by ion mobility to aid visual tracking across the mobility range. Text annotations report the number of matched precursors (n) and the corresponding median absolute deviation shown in each panel.

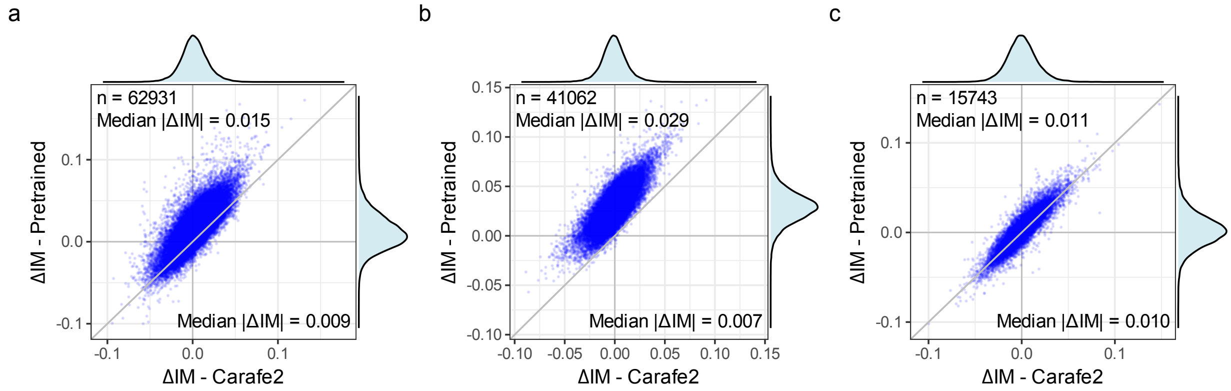

Supplementary Figure 3: **Comparison of ion mobility prediction between Carafe2 fine-tuned and pretrained models.** Scatter plots compare ion mobility prediction errors using Carafe2 fine-tuned models (x axis) and AlphaPeptDeep DDA data pretrained model (y axis) on the same three timsTOF DIA datasets used for Figure 3d–f, corresponding to panels **(a)**, **(b)**, and **(c)**, respectively. Text annotations report the number of matched precursors (n) and the corresponding median absolute prediction errors.

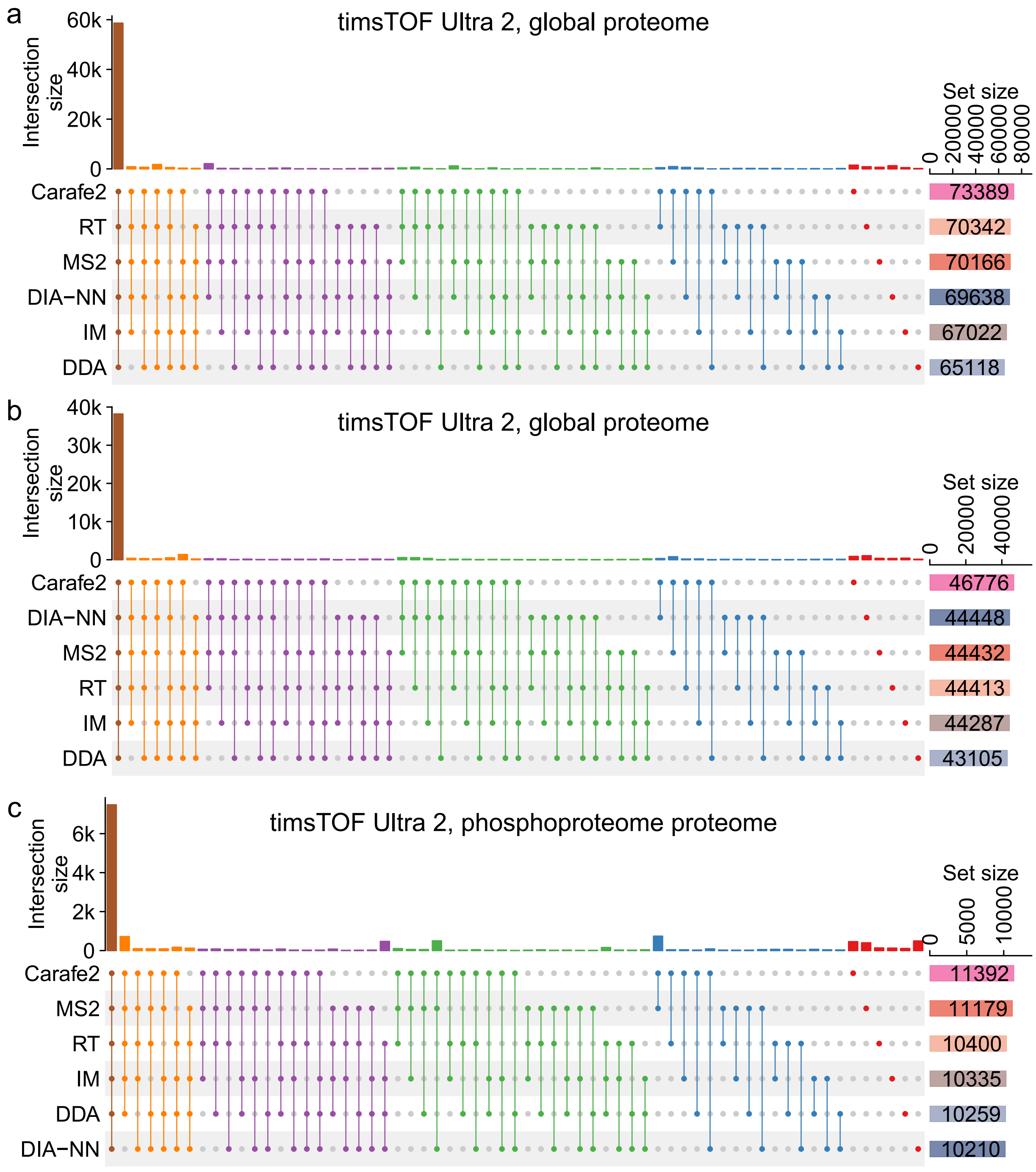

Supplementary Figure 4: **Upset plots showing the overlaps of peptide precursors detected using different *in silico* spectral libraries.** The upset plots correspond to the three datasets evaluated in Figure 4b–d of the main text: **(a)** a global proteome yeast dataset generated on a timsTOF Ultra 2 instrument, **(b)** a global proteome yeast dataset generated on a different timsTOF Ultra 2 instrument with a shorter LC gradient, and **(c)** a yeast phosphoproteome DIA dataset generated on a timsTOF Ultra 2 instrument. The dots representing combination sets are colored according to the number of sets included.

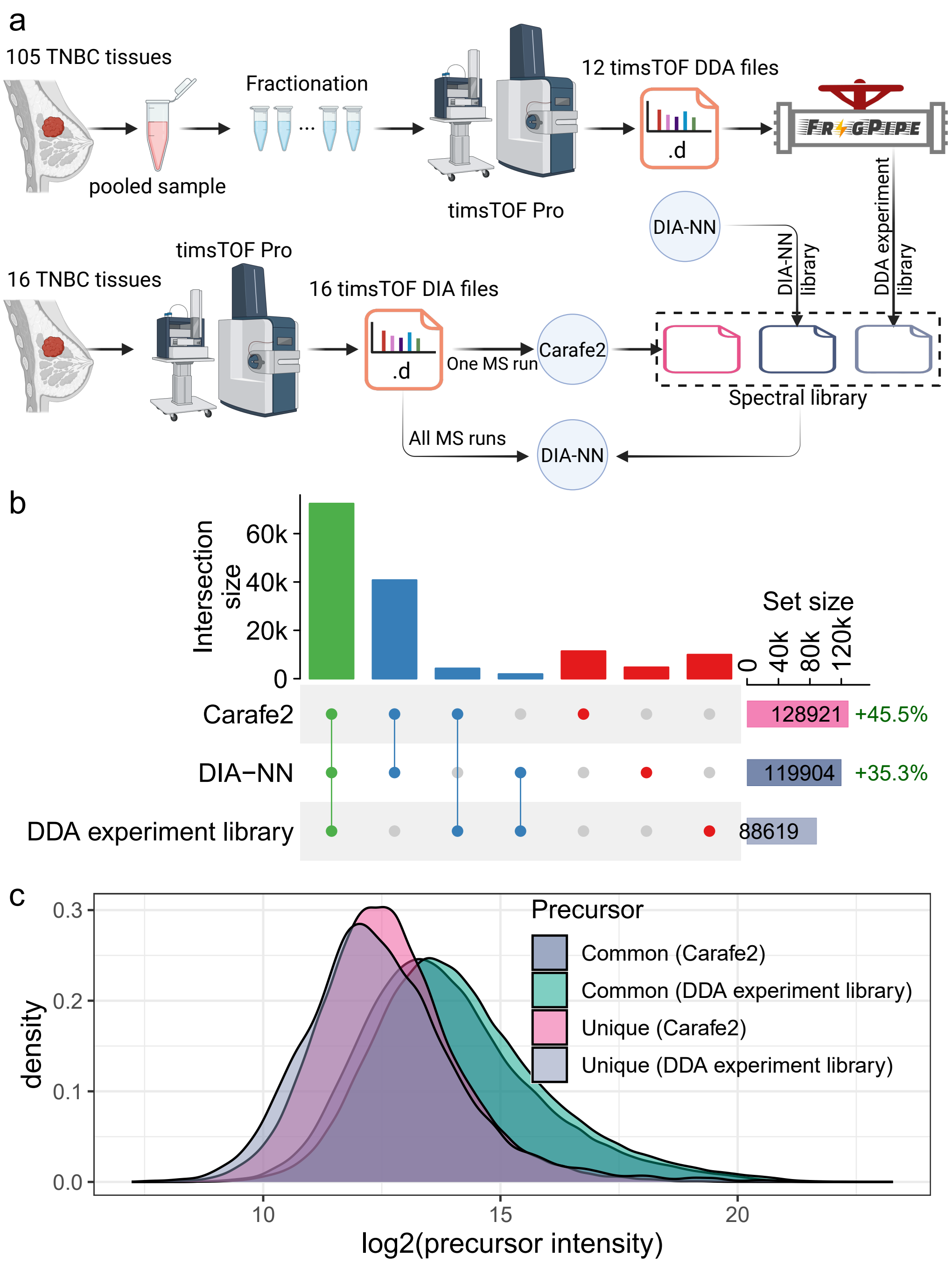

Supplementary Figure 5: **Comparison of *in silico* and DDA experimental spectral libraries. (a)** Comparing an experimental spectral library with *in silico* spectral libraries on a human tissue dataset. The DDA experimental spectral library was generated using FragPipe on 12 DDA timsTOF runs from the same study. The *in silico* spectral libraries were generated using DIA-NN and Carafe2, respectively. **(b)** An upset plot showing the number of precursors detected using different spectral libraries. **(c)** A precursor intensity density plot showing the distribution of precursor intensities for precursors detected between Carafe2 and DDA experiment library. Panel **(a)** was created using BioRender.
